# Clinical Implementation of NGAL Testing to Improve Diagnostic Assessment of AKI Episodes in a Canadian Center

**DOI:** 10.1177/20543581221118991

**Published:** 2022-08-17

**Authors:** Jean-Maxime Côté, Roxanne Authier, Isabelle Ethier, Jean-François Cailhier, William Beaubien-Souligny, Patrick T. Murray, Pierre-Olivier Hétu, Marie-Claire Bélanger

**Affiliations:** 1 Division of Nephrology, Department of Medicine, Centre hospitalier de l’Université de Montréal, QC, Canada; 2 Research Center (CRCHUM), Centre hospitalier de l’Université de Montréal, QC, Canada; 3 School of Medicine, University College Dublin, Ireland; 4 Division of Clinical Biochemistry, Centre hospitalier de l’Université de Montréal, QC, Canada

**Keywords:** NGAL, acute kidney injury, neutrophil gelatinase-associated lipocalin, diagnosis, nephrology, biomarker

## Abstract

**Background:**

The differential diagnosis of acute kidney injury (AKI) episodes is often challenging. Novel AKI biomarkers have shown their utility to improve prognostic prediction and diagnostic assessment in various research populations but their implementation in standard clinical practice is still rarely reported.

**Objective:**

To report the differential diagnostic ability and associated clinical utility of the neutrophil gelatinase-associated lipocalin (NGAL) testing in a real-life setting of a heterogeneous AKI population.

**Design:**

This is a retrospective cohort study combined with a clinical audit using questionnaires distributed to consultant nephrologists following NGAL results.

**Setting:**

The first 250 consecutive patients with a confirmed AKI where an NGAL test (plasma NGAL [pNGAL] or urine NGAL [uNGAL]) was ordered from a large academic center in Montreal, Canada from January 2021 to August 2021.

**Patients:**

Patients were classified into 3 groups based on the final AKI etiology category (functional, intrarenal, and postrenal) following definitive adjudication by 2 independent nephrologists.

**Methods:**

The ability of plasma NGAL (pNGAL), urine NGAL (uNGAL), and uNGAL-to-creatinine ratio (uNGAL/Cr) to discriminate intrarenal from functional AKI etiologies was compared to standard urine chemistry (FENa) and proteinuria. A logistic regression was used to evaluate the association between intrarenal AKI and increased biomarker levels. The overall clinical utility and appreciation of the NGAL test was evaluated using a questionnaire completed prospectively by the consultant nephrologist at the time of receiving the NGAL result. The NGAL results were prospectively available to clinicians with a median time of 2.9 (1.3-7.4) hours from the initial order.

**Results:**

A total of 214 uNGAL and 44 pNGAL were ordered from 100 functional, 139 intrarenal and 11 postrenal AKI episodes after final adjudication. The discriminative ability of FENa (AUC 0.68 [95% CI: 0.61-0.75]) was lower than uNGAL (AUC 0.80 [95% CI: 0.73-0.86]) and uNGAL/Cr (AUC 0.83 [95% CI: 0.77-0.88]) but better than pNGAL (AUC 0.66 [95% CI: 0.48-0.85]). According to consultant nephrologists, the NGAL testing has led to a change in clinical management in 42% of cases.

**Limitations:**

Data reported came from a single center and NGAL was reserved for more complex cases, which limits generalizability. No biopsy has been performed for most AKI cases as the final adjudication was based on a retrospective review of the hospitalization episode.

**Conclusions:**

Neutrophil gelatinase-associated lipocalin testing can be successfully integrated as part of the diagnostic workup for AKI in clinical practice. The integration of tubular damage biomarkers to functional biomarkers can further improve the differential diagnostic assessment. However, the impact of such biomarkers on AKI management and associated outcomes still needs further validation.

## Abrégé

**Contexte:**

Le diagnostic différentiel des épisodes d’insuffisance rénale aiguë (IRA) pose souvent un problème. De nouveaux biomarqueurs d’IRA ont montré leur utilité pour améliorer la prédiction pronostique et l’évaluation diagnostique dans diverses populations de recherche, mais leur application dans la pratique clinique est encore peu rapportée.

**Objectif:**

Rendre compte de la capacité de diagnostic différentiel et de l’utilité clinique du test NGAL (neutrophil gelatinase-associated lipocalin) dans le contexte réel d’une population hétérogène de patients atteints d’IRA.

**Devis:**

Étude de cohorte rétrospective combinée à un audit clinique mené par l’entremise de questionnaires distribués aux néphrologues consultants à la suite du résultat NGAL.

**Cadre:**

Les 250 premiers patients consécutifs avec une IRA confirmée, pour qui un test NGAL (plasmatique [pNGAL] ou urinaire [uNGAL]) avait été demandé entre janvier et août 2021 dans un grand centre universitaire de Montréal (Canada).

**Sujets:**

Les patients ont été classés en 3 groupes selon la catégorie étiologique finale de l’IRA (fonctionnelle, intrarénale, post-rénale) après révision par deux néphrologues indépendants.

**Méthodologie:**

La capacité du pNGAL, du uNGAL et du rapport uNGAL et du rapport uNGAL sur créatinine (uNGAL/Cr) à discriminer les étiologies fonctionnelles des étiologies intrarénales a été comparée à celle des indices urinaires standard de l’urine (FENa) et de la protéinurie. Une régression logistique a servi à évaluer l’association entre l’IRA intrarénale et la hausse des taux des biomarqueurs. L’appréciation du test NGAL et son utilité clinique globale ont été évaluées à l’aide d’un questionnaire rempli prospectivement par le néphrologue consultant lors de la réception du résultat NGAL. Les résultats NGAL ont été mis à la disposition des cliniciens de manière prospective, dans un délai médian de 2,9 [1,3-7,4] heures suivant la prescription initiale.

**Résultats:**

En tout, après la révision finale, 214 tests uNGAL et 44 tests pNGAL ont été demandés à partir de 100 épisodes d’IRA fonctionnelle, 139 épisodes d’IRA intrarénale et 11 épisodes d’IRA post-rénale. La capacité discriminante du FENa (SSC: 0,68 [IC 95 %: 0,61-0,75]) était inférieure à celles du uNGAL (SSC: 0,80 [IC 95 %: 0,73-0,86]) et du rapport uNGAL/ Cr (SSC: 0,83 [IC 95 %: 0,77-0,88]), mais supérieure à celle du pNGAL (SSC: 0,66 [IC 95 %: 0,48-0,85]). Les néphrologues ont indiqué que les tests NGAL avaient entraîné un changement dans la prise en charge clinique dans 42 % des cas.

**Limites:**

Les données provenaient d’un seul centre et le test NGAL était réservé aux cas plus complexes, ce qui limite la généralisabilité. Dans la plupart des cas, aucune biopsie n’a été effectuée et le diagnostic final était basé sur un examen rétrospectif de l’hospitalisation.

**Conclusions:**

En pratique clinique, les tests NGAL peuvent être intégrés avec succès au diagnostic de l’IRA. L’intégration des biomarqueurs de lésions tubulaires aux biomarqueurs fonctionnels peut améliorer davantage l’évaluation du diagnostic différentiel. Cependant, l’impact de ces biomarqueurs sur la prise en charge de l’IRA et les résultats connexes doit encore être validé.

## Introduction

Acute kidney injury (AKI) is a frequent complication in hospitalized patients, occurring in up to 20% of all hospitalisations^1^ and can affect more than 55% of patients admitted to the intensive care unit.^2^ Diagnostic and staging criteria for AKI occurrence is based on serum creatinine elevation and/or urine output reduction using KDIGO-AKI criteria,^3^ while the differential diagnosis is usually based on a combination of clinical factors and biologic markers. Earlier recognition of the exact cause may help clinicians to optimize AKI management in a timely manner, improve clinical outcomes such as renal recovery and possibly reduce costs associated with hospital-acquired AKI.^4,5^ This is especially true in patients with clinical syndromes requiring more targeted therapies than standard AKI supportive care, such as diuretics for cardiorenal syndromes (CRS), vasopressors plus intravenous albumin for hepatorenal syndromes (HRS) or, more frequently, intravenous fluid therapy for hypovolemic AKI.

The last 15 years have seen the emergence of novel kidney injury biomarkers to improve the prognostic and diagnostic assessment in patients with confirmed AKI and in those at risk of progression. Numerous studies, from various AKI populations, have repeatedly confirmed the usefulness of such AKI biomarkers to better recognize the different pathological processes involved, leading to an optimization in etiological diagnostic discrimination and accurate differential diagnosis.^6^ Indeed, the presence of tubular damage biomarkers such as the neutrophil gelatinase-associated lipocalin (NGAL) is clinically associated with intrinsic AKI, mostly acute tubular injury, while its absence is more in favor of functional (prerenal) causes.^7,8^ Recently, the 23nd *Acute Disease Quality Initiative* Consensus Conference recommended that a combination of functional and damage biomarkers should now be integrated with clinical information, to identify high-risk patients and to improve diagnostic assessment and management of AKI episodes in various clinical settings.^9^

Based on a favorable experience from a previous clinical implementation of NGAL testing in a tertiary hospital in Ireland,^8^ our group decided to implement NGAL testing at the *Centre hospitalier de l’Université de Montréal* (CHUM), the second largest Canadian hospital, with more than 800 beds, as a diagnostic tool for new-onset AKI episodes where uncertainties remain regarding the suspected cause at the time of nephrology consultation. This constitutes a large and comprehensive report on the clinical experience and associated biomarker accuracy with a combination of traditional and novel AKI biomarkers and the first to report a real-life setting clinical implementation and experience using NGAL testing in Canada.

## Methods

This study reports the results of a comprehensive clinical audit performed on the first 250 consecutive AKI cases at the CHUM where at least one NGAL test was ordered. The use of the NGAL was restricted to the nephrology consultation service for this implementation phase and was suggested as part of the initial diagnostic assessment in patients with already confirmed AKI, but in whom the definitive AKI etiology remains unclear or suspected to be multifactorial. Interpretation and subsequent management were left to the attending nephrologist. Thirteen different consultant nephrologists were involved in the order at least one NGAL test for these 250 AKI cases. The result of the NGAL was reported as all other clinical biochemistry results on the CHUM electronic medical record (EMR) as soon as analyzed by our local biochemistry laboratory. Urine NGAL (uNGAL) and plasma NGAL (pNGAL) could be measured, but uNGAL was generally preferred based on previous experiences showing a lower specificity and overall accuracy for pNGAL in hospitalized patients with already confirmed AKI.^8^ The NGAL was validated and measured using an automated AU5800 Chemistry Analyzer (BeckmanCoulter^©^) with the NGAL Test™ by BioPorto Diagnostics^©^ (Hellerup, Denmark) by turbidimetric immunoassay. The limit of quantification and measurable ranges for both uNGAL and pNGAL were 25 to 3000 ng/mL. The cost of NGAL testing, once implemented, was around 21.19 CAD$ per test. No clear cut-off point was reported with the result, but clinicians were trained at the time of integration and were instructed that ≥150 ng/mL was compatible with the presence of tubular damage in previous cohorts. The test was first made available at the CHUM in January 2021. Ordering of uNGAL testing was integrated to a comprehensive urine panel that included sodium, creatinine, albumin, protein, and a standard urine analysis (dipstick). In our lab, a reactive manual microscopy was automatically performed by a lab technician in case of any urine abnormalities. This clinical information was considered essential as urinary tract infection or even contamination with leukocyturia are significant causes of false positive uNGAL elevation and should be interpreted in that context. The EMR automatically reported the uNGAL over urine creatinine ratio (uNGAL/Cr) converted to ng/mg.

The use of an automated analyzer minimizes the delay between the initial order and the availability of the result, with a median time of 2.9 (1.3-7.4) hours, and also facilitates availability of the test during nights and on weekends; 236 from the 250 NGAL (94%) results were available within less than 24 hours.

The audit was designed to evaluate the ability of the NGAL test to discriminate intrarenal AKI episodes from functional causes (hypovolemia, CRS, HRS) in a real-life and heterogeneous clinical practice setting (primary objective). In addition, for all AKI cases where an NGAL test was ordered, the consultant nephrologist was asked to complete a short questionnaire regarding the objectives of the NGAL prescription in that context, its correlation with the suspected AKI etiology and to state if the NGAL result led to significant changes in AKI management. This short survey was accessible using a computer or a smartphone and was entirely anonymous. The nephrologist leading the implementation of the NGAL testing in our center was not involved in the order of any of these 250 first NGAL tests and did not answer any of these post-NGAL appreciation questionnaire.

The final adjudication of AKI categories (prerenal, intrarenal, postrenal) and final pre-defined causes (toxic ATN, ischemic ATN, glomerulonephritis [GN], tubulointerstitial nephritis, hypovolemia, CRS, HRS and postrenal) was based on chart review and was independently performed by two nephrologists who were not involved in the care of these patients and were blinded from NGAL results. The primary consensus was based on an anonymized and comprehensive dataset, which included daily urea and creatinine levels from 48 hours before the NGAL to 14 days after, conventional urine chemistry biomarkers, radiologic exam results, signs of systemic or urinary tract infections, as well as a detailed summary of the clinical case. An agreement was obtained at that first step for 236 cases (94%), then a final consensus was obtained through mutual discussion based on a comprehensive chart review of the remaining 14 cases. The blinding on all NGAL levels was maintained for all 250 cases during the entire adjudication process for one reviewer but was only partial for those complex AKI cases for the second reviewer, as some clinicians had mentioned the NGAL result in their clinical note revealing the result. At the end of the process, no disagreement persisted between both adjudicator nephrologists. This approach of adjudication is in accordance with standard clinical practice, as a kidney biopsy does not have to be performed in most AKI cases.

All descriptive statistics were reported as median with interquartile range and proportions. A Kruskal-Wallis nonparametric test (significance = .05) was used to compare the distribution of the median from each group for all AKI biomarkers (Fractional excretion of sodium [FENa], Urine sodium, pNGAL, uNGAL, uNGAL/Cr, urine albumin-to-creatinine ratio [uAlb/Cr], and urine protein-to-creatinine ratio [uProt/Cr]). The assessment of the diagnostic accuracy of all AKI biomarkers considered the clinical question “Does my patient with AKI have an intrarenal cause?.” First, the accuracy was evaluated using sensitivity, specificity, positive predictive value (PPV), negative predictive value (NPV) and the area under the receiver operating characteristic (AUROC) for all biomarkers individually. The optimal cut-off value was based on the Youden Index (representing the sum of sensitivity and specificity-1) determined from the overall cohort. In addition, positive and negative likelihood ratio (LR) and post-test probability of having an intrarenal AKI in presence of elevated uNGAL or uNGAL/Cr values were reported for the entire cohort as well as when considering the FENa validity. Finally, an exploratory logistic regression was used to evaluate the odds of facing an intrarenal AKI for every increases of 1% for FENa, 10 ng/mL for uNGAL, 10 ng/mg for uNGAL/Cr, 10 ng/mL for pNGAL, 0.01 g/mmol for uProt/Cr, and 10 mg/mmol for uAlb/Cr, as well as when adjusted for age, presence of chronic kidney disease (CKD), potential urine contamination, and recent diuretics exposure (<24 h). All statistical analyses were completed with SPSS 27.0 (IBM Corp^©^; Armonk, NY). This audit was pre-approved as part of a biomarker clinical implementation process, which granted a waiver of informed consent and local research ethics committee approval.

## Results

### Patient Characteristics

In patients with new-onset AKI who received NGAL testing, 174 (70%) of all episodes occurred in males, while the median age was 67.5 (58.0-74.0) years old. As shown in Table 1, 54 patients (22%) were hospitalized in the ICU, 41 (16%) were seen in the emergency room, and 3 (1%) in the outpatient clinic, while the remaining were hospitalized on regular wards. Regarding comorbidities, 40% of all patients had CKD (eGFR < 60 mL/min), and a significant proportion were either severely immunocompromised or transplanted (17%). Patients had various baseline comorbidities as depicted in Table 1.

**Table 1.** Baseline Characteristics.

Variable	Results (n = 250)
Median age, y	67.5 (58.0-74.0)
Male sex (%)	174 (70)
Hospitalization type (%):	
Medical	148 (59)
Surgical	96 (38)
ICU	54 (22)
Emergency room	41 (16)
Outpatient clinic	3 (1.2)
Comorbidities (%):	
Hypertension	155 (62)
Diabetes	100 (40)
Cirrhosis or acute liver disease	45 (18)
Heart failure	80 (32)
HfpEF	28 (11)
HfrEF^a^	52 (21)
Immunocompromised^b^ or transplanted	43 (17)
COVID-19^c^	4 (1.6)
CKD at baseline	101 (40)
eGFR < 30 mL/min/1.73 m^2^	24 (9.6)
Median eGFR at baseline, mL/min/1.73 m^2^	72 (45-89)
Hospital length of stay, days	20 (9-36)
In-hospital mortality (%)	52 (21)

*Note*. ICU = intensive care unit; HfpEF = heart failure with preserved ejection fraction; HfrEF = heart failure with reduced ejection fraction; CKD = chronic kidney disease (<60mL/min/1.73m)^2^; eGFR = estimated glomerular filtration rate; COVID-19 = coronavirus disease 2019.

a Defined as a Left ventricular ejection fraction ≤ 35%.

b Defined as chronic steroid, calcineurin inhibitor, anti-metabolite exposure or active chemotherapy.

c Defined as any PCR positive result during the hospitalization stay, with or without associated symptoms.

### AKI Episode Characteristics

At the time of NGAL measurement, the median serum creatinine was 229 (172-327) umol/L, while stage 1, 2, and 3 KDIGO-AKI occurred in 99 (40%), 58 (23%), and 93 (37%), respectively. Kidney replacement therapy was ongoing or initiated within 24h at the time of NGAL prescription for 16 (6%) patients. Importantly, urine contamination with either a confirmed urinary tract infection, asymptomatic bacteriuria, ileal pouch or significant leukocyturia occurred in 109 (44%) patients. Final adjudication resulted in 100 prerenal, 139 intrarenal, and 11 postrenal AKI episodes (Table 2). Additional characteristics from those episodes are reported in Supplemental Table S1 (Supplemental Material), and all biomarker results reported by final AKI etiologies are shown in Supplemental Table S2.

**Table 2.** Biomarkers Results According to the Final Acute Kidney Injury (AKI) Categories.

Biomarkers	n	All (n = 250)	Prerenal (n = 100)^a^	Intrarenal (n = 139)	Postrenal (n = 11)	*P* value^b^
pNGAL, ng/mL	44	324 (173-523)	238 (112-485)	363 (224-706)	235 (123-235)	.154
uNGAL, ng/mL	214	205 (48-914)	62 (25-172)	550 (191-1873)	60 (26-1556)	<.001
uNGAL/Cr, ng/mg	213	304 (68-1520)	71 (39-215)	917 (282-2715)	123 (36-1984)	<.001
Urine Sodium, mmol/L	243	38 (15-65)	30 (12-55)	42 (20-76)	49 (29-67)	.021
FENa, %	242	1.04 (0.37-2.79)	0.56 (0.25-1.57)	1.69 (0.52-3.99)	1.45 (1.04-1.96)	<.001
Ualb/Cr, mg/mmol	220	16 (5.0-47)	9.1 (2.8-28)	23 (7.5-58)	26 (1.7-115)	<.001
Uprot/Cr, g/mmol	232	0.08 (0.03-0.20)	0.04 (0.02-0.09)	0.13 (0.05-0.26)	0.11 (0.02-0.36)	<.001

*Note*. NGAL = neutrophil gelatinase-associated lipocalin; pNGAL = plasma NGAL; uNGAL = urine NGAL; uNGAL/Cr = urine NGAL-to-creatinine ratio; FENa = fractional excretion of sodium; Ualb/Cr = urine albumin-to-creatinine ratio; uprot/Cr = urine protein-to-creatinine ratio.

a Prerenal cases include hypovolemia, cardiorenal and hepatorenal syndromes.

b Using a Kruskal-Wallis Nonparametric Test.

### Biomarker Results by AKI Category

As shown in Table 2 and Supplemental Figure S1, a total of 214 uNGAL and 44 pNGAL were measured in the first 250 patients, while the FENa was available for 242 of those patients. No significant difference was observed across the three AKI categories for pNGAL values (*P* = .154), but there was a trend toward a higher median value in the intrarenal group (363 [224-706] ng/mL) compared to prerenal (238 [112-485] ng/mL). Urinary NGAL values were significantly higher for intrarenal AKI than prerenal or postrenal AKI, either for the uNGAL or the uNGAL/Cr ratio (*P* < .001). Notably, although the FENa was significantly higher in the intrarenal group (1.69 [0.52-3.99]%), the median value was still within the traditional diagnostic gray zone (between 1% and 2%). Functional AKI episodes were also associated with lower urine albumin- and protein-to-creatinine ratio than patients with intrarenal or postrenal AKI (*P* < .001).

### Biomarkers Accuracy to Discriminate Intrarenal From Functional AKI Episodes

In the overall cohort, the accuracy of FENa based on the standard 2% cut-off had a low sensitivity (46%) but relatively good specificity (81%) and PPV (76%), resulting in a modest but significant AUC (0.68 [95% CI: 0.61-0.75], *P* < .001). The presence of proteinuria was also discriminative of intrarenal AKI, especially the total protein-to-creatinine ratio (AUC = 0.75 [95% CI: 0.68-0.81], *P* < .001), where the Youden index (0.085 g/mmol, *equivalent to 0.75 g/g*) had specificity and PPV of 74 and 78% respectively. The overall discriminative ability of pNGAL was low with a sensitivity and specificity of both 67% and a non-significative AUC (0.66 [95% CI: 0.48-0.85], *P* = .081). On the opposite, the uNGAL (AUC = 0.80 [95% CI: 0.73-0.86], *P* < .001) had good discriminative accuracy, especially when considering the uNGAL over creatinine ratio (AUC = 0.83 [95% CI: 0.77-0.88], *P* < .001), where Youden indexes were, respectively, 139 ng/mL and 288 ng/mg. When compared to the standard FENa, only the uNGAL and uNGAL over creatinine ratio had a better discriminative performance based on AUC (respectively, *P* = .008 and *P* < .001).

### Integrating NGAL Testing Into the Diagnostic Assessment

**Figure 1.**
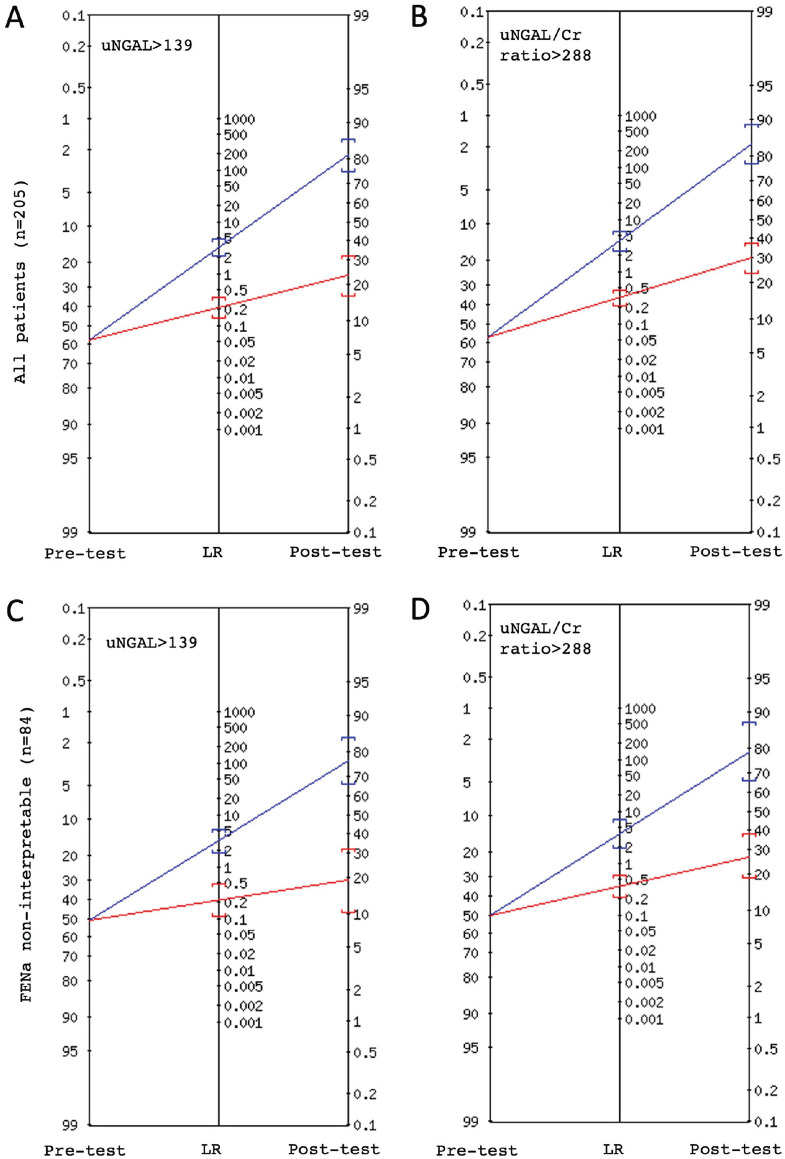
Fagan’s nomogram: change in the probability of intrarenal acute kidney injury (AKI) following the neutrophil gelatinase-associated lipocalin (NGAL) test result. (A) With uNGAL more than 139 ng/mL in patients with either intrarenal or functional AKI, (B) with uNGAL/Cr >288 ng/mg in patients with either intrarenal or functional AKI, (C) with uNGAL >139 ng/mL in patients having either intrarenal or function AKI and FENa result noninterpretable, (D) with uNGAL/Cr >288 ng/mL in patients with either intrarenal or functional AKI and FENa result noninterpretable. *Note*. Blue Line = positive result (>139 ng/mL or 288 ng/mg), Red Line = negative result (≤ 39 ng/mL or 288 ng/mL). LR = likelihood ratio; uNGAL = urine NGAL; uNGAL/Cr = urine NGAL-to-creatinine ratio; FENa = fractional excretion of sodium.

**Figure 2.**
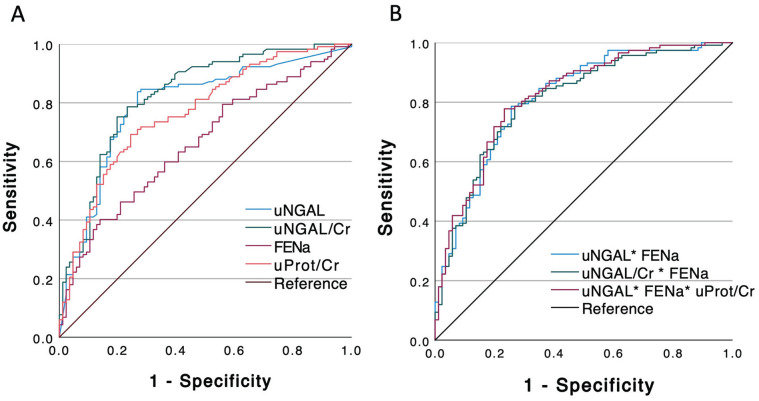
Receiver operating curve (ROC) representing the diagnostic ability of biomarkers to classify intrarenal acute kidney injury (AKI): (A) All biomarkers separately: uNGAL (blue line), uNGAL/Cr (green line), FENa (pink line), uProt/Cr (red line) and reference (black line), (B) combination of biomarkers: uNGAL+FENa (blue line), uNGAL/Cr+FENa (green line), uNGAL+FENa+uProt/Cr (pink line), reference (black line). *Note*. NGAL = neutrophil gelatinase-associated lipocalin; uNGAL = urine NGAL; uNGAL/Cr = urine NGAL-to-creatinine ratio; FENa = fractional excretion of sodium; uProt/Cr = urine protein-to-creatinine ratio.

As shown in Table 3 and Figures 1 and 2, for this AKI cohort, the presence of elevated uNGAL values increased the post-test probability of have an intrarenal cause to 81% with a positive LR of 3.1 [95% CI: 2.2-4.5]. Adjustment for urine concentration slightly improved the predictivity to 84% (+LR: 3.8 [95% CI: 2.5-5.9]), while low uNGAL and uNGAL/Cr levels were instead associated with improved negative LR. In this cohort, 78 patients had received a diuretic within the last 24 hours of the urine collection, leading to potentially uninterpretable FENa results. In addition, 37 patients had a FENa result within the gray interpretative zone (1%-2%). However, in that subgroup of patients where FENa was considered uninterpretable, the uNGAL and uNGAL/Cr kept their good discriminative ability (Figure 1 and Supplemental Table S3). The post-test probability and associated LRs of all other biomarkers are shown in Supplemental Figure S2.

**Table 3.** Accuracy of Diagnostic Biomarkers to Discriminate Intrarenal (n = 139) From Functional Acute Kidney Injury (n = 100) Episodes.

Biomarker	N	Results, AUC [95% CI]	AUC *P*-value^a^	AUC *P*-value^b^	Best cut-off^c^	Sensitivity	Specificity	PPV	NPV	+LR [95% CI]	−LR [95% CI]
FENa%	231	0.68 [0.61-0.75]	<.001	*REF*	>2%^d^	46	81	76	53	2.4 [1.5-3.8]	0.7 [0.6-0.8]
pNGAL	42	0.66 [0.48-0.85]	.081	.531	266 ng/mL	67	67	78	53	2.0 [0.9-4.3]	0.5 [0.3-1.0]
uNGAL	205	0.80 [0.73-0.86]	<.001	.008	139 ng/mL	84	73	81	77	3.1 [2.2-4.5]	0.2 [0.1-0.3]
uNGAL/Cr	204	0.83 [0.77-0.88]	<.001	<.001	288 ng/mg	75	80	84	70	3.8 [2.5-5.9]	0.3 [0.2-0.4]
uAlb/Cr	211	0.66 [0.59-0.73]	<.001	.893	19.6 mg/mmol	57	70	72	55	1.9 [1.4-2.7]	0.6 [0.5-0.8]
uProt/Cr	223	0.75 [0.68-0.81]	<.001	.081	0.085 g/mmol	67	74	78	63	2.6 [1.8-3.7]	0.5 [0.3-0.6]

*Note*. Based on final adjudication. AUC = area under the curve; CI = confidence interval; PPV = positive predictive value; NPV = negative predictive value; +LR = positive likelihood ratio; -LR = negative likelihood ratio; NGAL = neutrophil gelatinase-associated lipocalin; pNGAL = plasma NGAL; uNGAL = urine NGAL; uNGAL/Cr = urine NGAL-to-creatinine ratio; FENa = fractional excretion of sodium; uAlb/Cr = urine albumin-to-creatinine ratio; uProt/Cr = urine protein-to-creatinine ratio.

a Representing the *P* value of each AUC, measured under the nonparametric assumption.

b Representing the comparison of each biomarker AUC and to the FENa AUC (REF) using a paired-sample z-test.

c Using the Youden best cut-off index (sensitivity plus specificity-1).

d Corresponding to the traditional cut-off index used.

The accuracy to discriminate intrarenal from functional AKI episodes was slightly improved when combining the FENa to uNGAL testing (combined AUC: 0.81 [95% CI: 0.76-0.88]) but did not reach significance compared to uNGAL only (*P* = .432). Similarly, the combination of FENa to uNGAL/Cr (combined AUC: 0.81 [95% CI: 0.75-0.87]) did not improve the uNGAL/Cr accuracy (*P* = .279). A panel integrating uNGAL, FENa and uProt/Cr had a combined AUC of 0.82 [95% CI: 0.77-0.88] which did not outperform uNGAL used alone (*P* = .229; Figure 2).

### Association Between Biomarkers Level and Intrarenal AKI Diagnosis

As shown in Table 4, an increase by 1% of the FENa was associated with an increased risk of facing an intrarenal AKI (OR: 1.13 [95% CI: 1.07-1.19], *P* < .001) in the univariate analysis. That association was increased when adjusted for age, CKD and recent diuretics exposure (aOR: 1.35 [95% CI: 1.17-1.55], *p* < .001). Each increase of uNGAL by 10 units was associated with intrarenal AKI (OR: 1.01 [95% CI: 1.01-1.02], *P* < .001), with similar results when considering potential urine contamination, or adjustment for the urine creatinine (uNGAL/Cr ratio). On the opposite, there was no association between pNGAL elevation and intrarenal AKI (*P* = .518), despite adjustment for concomitant systemic infections (aOR: 1.01 [95% CI: 0.98-1.02], *P* = .590).

**Table 4.** Odds Ratio of Intrarenal Acute Kidney Injury (AKI) According to Variation in Biomarkers Levels.

Biomarker	N	Odds ratio [95% CI]	*P* value	Adj. odds ratio [95% CI]	*P* value
FENa, per 1% increase	205	1.13 [1.07-1.19]	<.001	1.35 [1.17-1.55]^a^	<.001
uNGAL, per 10 ng/mL increase	205	1.01 [1.01-1.02]	<.001	1.01 [1.01-1.02]^b^	<.001
uNGAL/Cr, per 10 ng/mg increase	204	1.01 [1.01-1.01]	<.001	1.01 [1.01-1.02]^b^	<.001
pNGAL, per 10 ng/mL increase	42	1.01 [0.99-1.02]	.518	1.01 [0.98-1.02]^c^	.590
uProt/Cr, per 0.01 g/mmol increase	223	1.07 [1.04-1.10]	<.001	1.07 [1.03-1.10]^d^	<.001
uAlb/Cr, per 10 mg/mmol increase	211	1.11 [1.03-1.20]	.005	1.11 [1.03-1.19]^d^	.009

*Note*. Using a logistic regression. CI = confidence interval; NGAL = neutrophil gelatinase-associated lipocalin; pNGAL = plasma NGAL; uNGAL = urine NGAL; uNGAL/Cr = urine NGAL-to-creatinine ratio; FENa = fractional excretion of sodium; uAlb/Cr = urine albumin-to-creatinine ratio; uProt/Cr = urine protein-to-creatinine ratio.

a Adjusted for: age, chronic kidney disease (CKD) and diuretics within 24 hours of urine sampling.

b Adjusted for: age, CKD, confirmed urinary tract injection, asymptomatic bacteriuria or leukocyturia >6 cells per microscopy field.

c Adjusted for: age, CKD, systemic infections.

d Adjusted for: age, CKD, diabetes, haematuria (at least 1+ on urine dipstick).

### Consulting Nephrologists’ Assessment of Clinical Utility in Practice

A response to the audit questionnaire for at least one question was available for 65 AKI episodes, representing an overall response rate of 26%, as shown in Table 5. These responses came from 9 different nephrologists. The NGAL testing as part of the initial nephrology management was considered relatively or highly useful for 45 (69%) AKI episodes. In most cases (95%), the NGAL was ordered for the purpose of differential diagnosis. Notably, nephrologists reported that the NGAL result has led to a change in the medical management for 42% of cases.

**Table 5.** NGAL Appreciation per AKI Episode by the Nephrology Consultation Service (n=65 Episodes).

Question	Result (%)
What was the clinical purpose when ordering the NGAL test? (n = 65)	
For diagnostic (yes)	62 (95%)
For pronostic (yes)	17 (26%)
Was the NGAL result in accordance with your preliminary diagnostic hypothesis? (Yes) (n = 65)	53 (82%)
Has the NGAL result led to a change in the clinical management of this AKI episode? (Yes) (n = 57)	24 (42%)
Please select the most appropriate option regarding your appreciation of the NGAL for its usefulness in the context of this AKI episode?	
1- Useless	2 (3%)
2- Low	5 (8%)
3- Moderate	13 (20%)
4- Relatively useful	24 (37%)
5- Highly useful (the result had changed clinical management)	21 (32%)

*Note*. NGAL = neutrophil gelatinase-associated lipocalin; AKI = acute kidney injury.

## Discussion

This report showed that novel AKI biomarkers can be successfully integrated into standard clinical diagnostic assessment. When considering all biomarkers individually, the accuracy of uNGAL and uNGAL/Cr to discriminate intrarenal AKI from functional causes surpassed FENa, albuminuria and proteinuria. Interestingly, the diagnostic accuracy of NGAL testing was maintained in presence of diuretics or even when FENa could not be interpretable (between 1% and 2%). The odds of facing an intrarenal AKI gradually progressed for each increase by 10 units of uNGAL (or uNGAL/Cr), as opposed to pNGAL, where no clear association could be identified. Overall, the access to NGAL testing was appreciated by consultant nephrologists.

Numerous cohort studies have reported the ability of early AKI biomarkers, such as TIMP-2*IGFBP7, KIM-1 or NGAL to predict AKI occurrence before creatinine elevation after renal insults such as cardiac surgery.^9^ Recent trials also showed that nephroprotective measures can be administered to patients with early signs of tubular damage (high AKI biomarkers with normal creatinine, especially TIMP2*IGFBP7) to minimize the risk of progression to AKI and severe AKI.^10-12^ These biomarkers have also shown their prognostic ability to predict the risk of progression to severe AKI, including KRT,^13,14^ and finally their usefulness for the differential diagnosis of AKI in a complex clinical syndrome.^7^ Optimizing the accuracy of the initial diagnostic assessment may help clinicians to minimize exposure of patients to incorrect treatments that may be ineffective or even harmful, such as empiric administration of additional intravenous fluids in normovolemic oliguric patients with ATN, resulting in detrimental fluid accumulation with associated comorbidities.^15^ Furthermore, some conditions may benefit from early specific intervention with therapies targeted to appropriate diagnoses, such as diuretics for CRS, or vasopressors plus intravenous albumin in patients with suspected AKI-HRS who failed to respond to initial intravenous fluid repletion, or immunosuppression for rapidly progressive glomerulonephritis.

Our local NGAL implementation and its associated audit was expressly designed to evaluate the ability of this novel AKI biomarker, and its combination with traditional urine chemistries, to help clinicians at bedside, when facing complex AKI cases, to optimize the differential diagnostic assessment in a real-life setting. Indeed, most of the literature on AKI biomarkers has reported data from relatively homogeneous research cohorts where results from these biomarkers were rarely available to attending physicians at the time of diagnosis. In contrast, the present study reports a real-life experience from heterogeneous patients where the result of the NGAL was quickly available to the clinician within the same day, as we consider this test characteristic essential for any acute diagnostic AKI biomarker used in clinical practice. This allows us to evaluate the overall appreciation of that new test by the clinician prospectively.

Some of the first 250 AKI episodes captured were classified as postrenal (n = 11). However, the role of the NGAL (and all other tubular damage biomarkers) as implemented in our center was mostly to discriminate intrarenal from functional AKI episodes, as postrenal AKI is managed differently, and such biomarkers have no clear relevance here. Following exclusion of postrenal cases, all associations and discriminative analyses were performed on the sub-cohort of patients with either intrarenal or functional AKI. For these 239 patients, we showed that the performance of the traditional FENa when used with the standard 2% cut-off point (suggesting intrarenal AKI) was relatively modest despite been widely used in practice. We also showed that adjusting the uNGAL for urinary concentration with the uNGAL-to-creatinine ratio improved the overall discrimination accuracy.

The determination of the optimal cut-off point for any diagnostic biomarker needs to balance the pretest probability and the cost of misdiagnosis. In the specific context of NGAL testing, a recent meta-analysis on NGAL prognostic abilities reported a high variability in cut-off values used in most studies, and that cut-off also varied according to the end-point of interest.^14^ However, no meta-analysis has specifically investigated the optimal cut-off when considering the NGAL as a discriminative tool for AKI differential diagnosis. In this study, using a standard statistical approach based on the Youden index, we found in our cohort an optimal cut-off value for the uNGAL (139 ng/mL) remarkably similar to the one generally reported in the most recent literature (150 ng/mL),^16^ and we reported a cut-off point for the uNGAL/Cr level (288 ng/mg) for the first time in a general AKI cohort, which still needs to be externally validated. Importantly, the Youden index has been criticized for its risk of overfitting and potentially overestimating performances.^17^ This might constitute a limitation of the current report despite obtaining cut-off levels (139 ng/mL) close to previously reported studies. As previously described, the pNGAL did not show clear utility in a clinical context with a broad spectrum of patients where concomitant systemic infections was relatively high. Based on these results, we decided to stop offering the pNGAL in our center, as the clinical usefulness and additive value compared to uNGAL has not been demonstrated.

This study also reported interesting data on the underestimated utility of albuminuria and proteinuria to corroborate the presence of tubular damage. Indeed, the association between proteinuria and glomerular disease is well known, but notable persistent ischemic and/or toxic tubular injury can lead to proximal tubular necrosis and local inflammation, reducing the capacity to reabsorb filtered albumin, while increasing the overall protein loss by the tubular degeneration/regeneration process.^18^ This sub-nephrotic protein loss was observed for none of the functional AKI cases (hypovolemia, CRS, HRS), where the tubular functions are normally preserved (Supplemental Table S2). The use of such readily available and easily measured biomarkers should not be underestimated when considering the differential diagnosis of intrarenal versus functional AKI, and not be limited to only rule-out glomerular diseases as part of the diagnostic process.

We decided to report the discriminative accuracy that leads to the final AKI diagnosis using 2 different methods. First, we reported the standard approach where the discriminative accuracy (to identify intrarenal AKI) was evaluated for all biomarkers separately, then when combining FENa to uNGAL and uProt/Cr, and when considering the presence of diuretics. We showed that, even in patients where FENa was noninterpretable, the uNGAL and uNGAL/Cr remain clinically useful. A second exploratory method showed a significant association between each increase of these novel biomarkers and the odds of facing an intrarenal AKI. Interestingly, that association was maintained in the adjusted model, similar to previously published data.^19^

This study was not designed to confirm the clinical benefit of NGAL testing in optimizing management and outcomes following an AKI episode. However, as shown by the post-NGAL questionnaire, the use of that novel biomarker in clinics was globally appreciated by consultant nephrologists. Indeed, in the caveat of a relatively low response rate, clinicians ordering the test mentioned that NGAL result has led to a change in the immediate management for a substantial proportion of patients (42%), despite being in accordance with their initial suspected diagnosis for most of them (82%). When questioning these consultants, most reported that the use of the NGAL has notably changed how their initial management, especially intravenous fluid therapy, was managed. As example, they reported a patient with heart failure treated with diuretics and suffering from a KDIGO stage 2 AKI following a late presentation of an already resolved viral gastroenteritis at the time of the nephrology consultation. In that case, a FENa more than 1% was uninterpretable. The presence of high level of NGAL (594 ng/mL) quickly confirmed the diagnosis of an ongoing ATN. Then, instead of continuing the trial of IV hydration for another 24h, the clinician was reassured by the diagnosis of ATN and quickly stopped the IV hydration, potentially preventing complications associated with positive fluid balance. Another nephrologist reported the case of a patient with AKI and decompensated cirrhosis who failed to response to initial intravenous albumin therapy, in whom the low uNGAL/Cr and low FENa 24 hours later oriented the clinician to quickly initiate the treatment of a functional hepatorenal syndrome with targeted therapies (ie. vasopressors). In both scenarios, the NGAL testing agreed with the suspected initial diagnosis, but its availability at bedside allow the clinician to quickly confirm the diagnosis and establish appropriate support in a timely manner. However, the current study was not designed to report such effect on the clinical management, nor on the overall cost-effectiveness.

This report has several limitations. No biopsy was performed for most patients, the final adjudication was therefore based on a comprehensive retrospective review of all AKI cases. Then, some patients might have been misclassified, with a trend toward the null hypothesis. In this real-life clinical implementation, the decision to order the NGAL test was made by the consulting nephrologist, who might has reserved the test for patients with more complex and severe AKI cases where a diagnostic uncertainty was still present. This aspect could limit the generalizability of these findings for less severe AKI cases. The timing between the first day of meeting KDIGO AKI criteria, the time of initial nephrology consultation and NGAL order varied across all AKI cases. Therefore, for some patients with late referral, the peak of NGAL elevation might have been missed, once again, leading toward the null hypothesis. In addition, infection-associated AKI (“*septic AKI”*) was not specially identified as a separated category for the final etiologic adjudication, some of them might have been classified either as toxic ATN, ischemic ATN or prerenal according to each episode’ characteristics. Neutrophil gelatinase-associated lipocalin testing being influenced by concomitant infections, it might have negatively affected discriminative accuracy results. Also, nephrologists answered the appreciation survey for only 65 of the 250 AKI cases, which brings a potential sampling bias. The lack of time was the principal reason given by clinicians for not completing the post-test survey.

This report confirms that NGAL testing, especially uNGAL and the uNGAL/Cr, can be routinely used in clinical practice as part of the workup for diagnostic assessment, especially for more complex AKI cases. Tubular damage biomarkers like the NGAL and functional biomarkers like the FENa could be used together to optimize the differential diagnosis, but should be interpreted in context of their own limitations, especially urinary tract infections and diuretics exposure respectively. Integration of the NGAL was valued by clinicians and some of them reported positive impacts when considering the initial AKI management. The impact of using tubular damage biomarkers on AKI-associated outcomes needs further validation.

## Supplemental Material

sj-docx-1-cjk-10.1177_20543581221118991 – Supplemental material for Clinical Implementation of NGAL Testing to Improve Diagnostic Assessment of AKI Episodes in a Canadian Center Click here for additional data file. Supplemental material, sj-docx-1-cjk-10.1177_20543581221118991 for Clinical Implementation of NGAL Testing to Improve Diagnostic Assessment of AKI Episodes in a Canadian Center by Jean-Maxime Côté, Roxanne Authier, Isabelle Ethier, Jean-François Cailhier, William Beaubien-Souligny, Patrick T. Murray, Pierre-Olivier Hétu and Marie-Claire Bélanger in Canadian Journal of Kidney Health and Disease
