# Supplementary material for: Clinical Implementation of NGAL Testing to Improve Diagnostic Assessment of AKI Episodes in a Canadian Center
Source: Can J Kidney Health Dis. 2022 Aug 17;9:20543581221118991. doi: 10.1177/20543581221118991 (PMC9393659; doi:10.1177/20543581221118991)
Supplement: sj-docx-1-cjk-10.1177_20543581221118991 – Supplemental material for Clinical Implementation of NGAL Testing to Improve Diagnostic Assessment of AKI Episodes in a Canadian Center [file sj-docx-1-cjk-10.1177_20543581221118991.docx]

**Supplementary Material**

Clinical Implementation of NGAL Testing to Improve Diagnostic Assessment of AKI Episodes in a Canadian Center

**Fig. S1**. Boxplots of Biomarker results according to the AKI category.

**Fig. S2.** Fagan’s Nomogram : Change in the probability of intrarenal AKI following testing.

1. *With FENa more than 2% in patients with either intrarenal of functional AKI, B) With pNGAL more than 266 ng/mL in patients with either intrarenal or functional AKI, C) With uProt/Cr> 0.085 g/mmol in patients with either intrarenal of functional AKI, D) With uAlb/Cr > 19.6 mg/mmol in patients with either intrarenal or functional AKI*

*LR: Likelihood ratio, Blue Line= Positive result, Red Line=Negative*

**Table S1.** AKI episode characteristics at the time of NGAL measurement

**Table S2.** Biomarkers results according to the final AKI etiology

**Table S3.** Discrimination ability of various biomarkers following FENa testing

Fig S1. Boxplots of Biomarker results according to the AKI category.

Fig S2. Fagan’s Nomogram : Change in the probability of intrarenal AKI following testing.

1. *With FENa more than 2% in patients with either intrarenal of functional AKI, B) With pNGAL more than 266 ng/mL in patients with either intrarenal or functional AKI, C) With uProt/Cr> 0.085 g/mmol in patients with either intrarenal of functional AKI, D) With uAlb/Cr > 19.6 mg/mmol in patients with either intrarenal or functional AKI*

*LR: Likelihood ratio, Blue Line= Positive result, Red Line=Negative*

| **Table S1. Acute Kidney Injury Episode Characteristics at the time of NGAL measurement** | |
| --- | --- |
| **Variables** | **Results (n=250)** |
| ***At the time of NGAL measurement*** | |
| Median serum creatinine, $\mu$mol/L | 229 (172-327) |
| Median serum urea, mmol/L | 19 (13-26) |
| Median serum albumin, g/L | 30 (27-34) |
| Median C-reactive protein, mg/L (n=139) | 94 (28-215) |
| KDIGO-AKI staging (%) |  |
| Stage 1 | 99 (40) |
| Stage 2 | 58 (23) |
| Stage 3 | 93 (37) |
| Undergoing acute KRT (%) | 16 (6) |
| Active urinary tract infection or Asx bacteriuria (%) | 30 (12) |
| Any urine contamination (%) | 109 (44) |
| Active sepsis (%) | 33 (13) |
| ***Overall AKI outcomes*** | |
| Maximum serum creatinine, $\mu$mol/L | 248 (181-384) |
| Maximum serum urea, mmol/L | 21 (14-30) |
| KRT initiated (%) | 44 (18) |
| *AKI; Acute kidney injury, Asx; Asymptomatic; KRT; Kidney replacement therapy* | |

| **Table S2. Biomarkers results according to the final AKI etiology** | | | | | | | | |
| --- | --- | --- | --- | --- | --- | --- | --- | --- |
|  | n | pNGAL, ng/mL | uNGAL, ng/mL | uNGAL/Cr, ng/mmol | U sodium, mmol/L | FENa% | uAlb/Cr, mg/mmol | uProt/Cr, g/mmol |
| *Prerenal* |  |  |  |  |  |  |  |  |
| Hypovolemia | 63 | 452 [256-641] | 55 [25-172] | 67 [39-218] | 32 [13-57] | 0.56 [0.27-1.48] | 6 [2-31] | 0.052 [0.015-0.098] |
| CRS | 27 | 116 [48-190] | 49 [25-168] | 85 [43-303] | 35 [13-59] | 0.76 [0.34-2.09] | 10 [5-21] | 0.044 [0.026-0.087] |
| HRS | 10 | 112 [112- ] | 74 [60-258] | 94 [38-451] | 12 [10-31] | 0.29 [0.13-1.00] | 11 [6-42] | 0.046 [0.027-0.106] |
| *Intrarenal* |  |  |  |  |  |  |  |  |
| Toxic ATN | 40 | 249 [221-362] | 449 [180-915] | 774 [200-2438] | 39 [13-70] | 1.33 [0.34-4.45] | 22 [8-51] | 0.116 [0.032-0.249] |
| Isch ATN | 84 | 468 [314-763] | 722 [228-2078] | 1133 [325-3158] | 44 [18-74] | 1.47 [0.52-3.62] | 21 [7-52] | 0.142 [0.053-0.261] |
| AIN | 5 | - | 277 [134-2807] | 1289 [402-6552] | 82 [61-99] | 4.00 [3.34-7.75] | 142 [27-902] | 0.500 [0.094-1.921] |
| GN | 7 | 271 [271- ] | 309 [75-710] | 701 [124-1901] | 38 [21-92] | 3.97 [0.67-7.76] | 98 [25-235] | 0.259 [0.110-0.706] |
| *Post-renal* |  |  |  |  |  |  |  |  |
| Any | 11 | 235 [123- 235] | 60 [26-1556] | 123 [36-1984] | 49 [29-67] | 1.45 [1.05-1.96] | 26 [2-115] | 0.114 [0.016-0.360] |

| **Table S3. Discrimination ability of various biomarkers following FENa testing** | | | | | | | | |
| --- | --- | --- | --- | --- | --- | --- | --- | --- |
| **Context** | **Biomarker (n)** | **Results, AUC [95% CI]** | **AUC p-value** | **Best cut-off** | **Sensi-tivity** | **Speci-ficity** | **PPV** | **NPV** |
| In AKI cases adequately classified with FENa | pNGAL (n=23) | 0.621 [0.382-0.860] | 0.325 | 266 ng/mL | 0.818 | 0.583 | 0.643 | 0.778 |
|  | uNGAL (n=104) | 0.773 [0.680-0.867] | <0.001 | 139 ng/mL | 0.808 | 0.750 | 0.764 | 0.796 |
|  | uNGAL/Cr (n=104) | 0.888 [0.826-0.951] | <0.001 | 288 ng/mg | 0.808 | 0.846 | 0.840 | 0.815 |
|  |  | | | | | | | |
| In AKI cases misclassified with FENa | pNGAL (n=19) | 0.833 [0.612-1.00] | 0.074 | 266 ng/mL | N/A |  |  |  |
|  | uNGAL (n=101) | 0.807 [0.714-0.900] | <0.001 | 139 ng/mL | 0.866 | 0.706 | 0.853 | 0.727 |
|  | uNGAL/Cr (n=100) | 0.746 [0.639-0.854] | <0.001 | 288 ng/mg | 0.712 | 0.735 | 0.839 | 0.568 |
|  |  | | | | | | | |
| In AKI cases misclassified with FENa AND no sign of urine contamination | pNGAL (n=6) | N/A |  |  |  |  |  |  |
|  | uNGAL (n=59) | 0.848 [0.747-0.948] | <0.001 | 139 ng/mL | 0.750 | 0.870 | 0.900 | 0.690 |
|  | uNGAL/Cr (n=58) | 0.801 [0.685-0.918] | <0.001 | 288 ng/mL | 0.628 | 0.913 | 0.917 | 0.612 |
